# Intestinal autonomy in extreme premature infants with short bowel syndrome: national experience

**DOI:** 10.1007/s00383-025-06036-4

**Published:** 2025-05-24

**Authors:** Saleem Mammoo, Noora Alshahwani, Maraeh Angela Mancha, Hassan Baghazal, Mansour Ali, Guy Brisseau

**Affiliations:** General and Thoracic Surgery Department, Sidra Medicine Doha, Doha, Qatar

**Keywords:** Premature, Short bowel syndrome, Bowel expansion, STEP

## Abstract

**Purpose:**

To investigate the outcomes of premature neonates with short bowel syndrome (SBS) managed under a BEAR (Bowel Elongation and Advanced Rehabilitation) protocol.

**Methods:**

This was a retrospective cohort study of preterm patients with SBS, treated at Sidra Medicine between January 2018 and February 2024. Data were extracted from electronic medical records, including patient demographics, clinical history, surgical interventions, parenteral nutrition (PN) duration, and long-term outcomes. The BEAR protocol incorporated a multidisciplinary approach with structured intestinal rehabilitation, hepato-protective PN strategies, and staged surgical interventions to promote enteral autonomy.

**Results:**

A total of 20 premature neonates with SBS were analyzed, with a median gestational age of 28 weeks and a median birth weight of 860g. Necrotizing enterocolitis was the primary cause of SBS in 90% of cases. Of the cohort, 80% successfully weaned off PN, achieving enteral autonomy at a median corrected age of 19.7 months. Seven patients underwent serial transverse enteroplasty (STEP), with 6/7 successfully transitioning to full enteral feeding. The study demonstrated favorable survival rates and reduced PN-associated complications.

**Conclusion:**

The BEAR protocol provides a structured and effective approach to intestinal rehabilitation in premature neonates with SBS, facilitating early enteral autonomy and minimizing long-term PN dependence. These findings contribute valuable clinical insights into optimizing multidisciplinary management strategies for this high-risk population.

## Introduction

Short bowel syndrome (SBS) is a debilitating multi-systemic disease resulting from the surgical resection of intestines, either due to congenital defects or acquired diseases. This condition often leads to long-term dependence on parenteral nutrition (PN), which introduces its own set of complications and challenges, particularly in preterm patients. Achieving intestinal autonomy, defined as the ability to maintain adequate nutrition and hydration through enteral feeding alone, is the primary aim of therapy for these patients [1]. Success in this area promises a life free of PN and its associated long-term complications. Infants are unique in this regard as they have improved chances at adaptation [2].

The incidence of SBS in very low birth weight (VLBW) infants at the National Institute of Child Health and Development (NICHD) neonatal research network centers was 0.7% (89 out of 12,316 live births).

between 2002 and 2005 [3]. A study from Canada revealed that SBS affects approximately 24.5 out of every 100,000 live births [4]. The incidence was notably higher in preterm infants, with 353.7 cases per 100,000 live births, while full-term infants had a much lower rate of 3.5 per 100,000 [4]. With significant advances in care, recent data show intestinal autonomy rate of 76% can be achieved when followed up in adolescents [5]. Similarly, Petit et al. reported a 73% weaning-off rate from home parenteral nutrition (HPN), with a median weaning time of 21 months, emphasizing the critical role of anatomical factors such as remnant bowel length (> 40 cm), the presence of the ileocecal valve (ICV), and retention of more than 50% of the colon [23]. Furthermore, the authors documented an impressive 98% 10-year survival rate in a cohort with a median PN duration of 10 months, setting a new target for professionals caring for patients with this condition [24].

In our unit, we employ a multifaceted approach to managing short bowel syndrome (SBS), integrating advanced techniques that optimize patient outcomes. At the core of our strategy is the autologous gastrointestinal reconstruction, a surgical technique designed to maximize bowel length and improve function. This procedure, combined with hepato-protective PN, careful venous preservation, and long-term multidisciplinary follow-up, serves as the foundation of our specialized bowel expansion and autologous reconstruction (BEAR) Program. BEAR is a comprehensive program designed to facilitate a gradual transition to intestinal autonomy while improving the overall quality of life for these vulnerable infants. By addressing both the medical and surgical needs of SBS patients, we aim to support long-term intestinal adaptation and independence from PN.

This study aims to summarize outcomes in premature neonate with SBS and assess the effect of following a regimented BEAR intestinal rehabilitation protocol as a baseline for future studies.

## Methods

This retrospective study examined preterm patients diagnosed with short bowel syndrome (SBS) who received treatment at Sidra Medicine between January 2018 and February 2024. Ethical approval was obtained from the Institutional Review Board (IRB) before initiating the study. IRB NUMBER: 1,865,534 and SDR NUMBER: 600,164.

### Inclusion and exclusion criteria

We included infants born at ≤ 32 weeks gestation who were either born at Sidra Medicine or referred to Sidra Medicine for further management. Patients were excluded if they had intestinal failure not related to SBS, had passed away prior to receiving care at Sidra, or were initially seen at Sidra but sought surgical treatment abroad with no local management or intervention. We also excluded patients with inadequate data on the chart (referred from another hospital).

### Intestinal rehabilitation protocol

Our approach followed the protocols established by the Manchester Intestinal Rehabilitation Program; a multi-disciplinary approach tailored for managing SBS in preterm infants [8]. Key elements include hepatic-sparing PN to minimize liver damage, careful central venous access management to prevent infections and loss of venous access, and reconstructive intestinal surgery when indicated to optimize bowel function. Additionally, we address the social and developmental needs of patients to support holistic growth and well-being.

1) Surgical intervention:

Surgical interventions, when required, involved controlled bowel expansion using proximal and distal tube stomas [9]. This is done mainly in cases with shorter bowel length anticipated to have a longer need for PN. This technique facilitates bowel growth and adaptation by introducing intermittent obstruction. It also allows for bowel recycling with distal refeeding. The goal is to maximize intestinal length and function, thereby reducing long-term PN dependence. serial transverse enteroplasty (STEP) was employed whenever the bowel diameter reached >=4 cm in an otherwise well baby. Beyond that, there were no set criteria to perform STEP, and it was left to the discretion of the treating surgeon.

Patients who did not undergo serial transverse enteroplasty (STEP) were those who had adequate residual bowel length (40 cms or more with an IC Valve) following their initial surgical resection or at the time of stoma closure and, therefore, did not require any form of bowel lengthening (101). These patients primarily underwent stoma closure as the only surgical intervention beyond their initial resection. Despite having sufficient bowel length, they required prolonged total parenteral nutrition (TPN) due to intestinal adaptation challenges before achieving enteral autonomy.

2) Parenteral nutrition:

SMOF (soy, medium-chain triglyceride, olive, and fish oil) lipid is the first-line lipid emulsion for all patients. Lipids are initiated at a safe starting dose, with defined maximum limits (1-3g/kg/d). If IFALD develops, lipid reduction strategies are implemented (limit to 1g/kg), and in severe or refractory cases, lipids may be completely restricted. Lipid reintroduction is carefully monitored, ensuring nutritional needs are met while minimizing hepatotoxicity. In cases where intestinal failure-associated liver disease (IFALD) is established and bilirubin levels remain persistently elevated despite interventions—such as reducing or completely with holding SMOFlipid—Omegaven (100% fish oil) is used to provide essential fatty acids for nutritionally deficient children.

We classify children into risk groups based on disease etiology and their likelihood of developing IFALD, as outlined in our PN guidelines. High-risk patients receive individualized lipid and carbohydrate regimens, with close monitoring of bilirubin levels, liver enzymes, and growth parameters to ensure early intervention and optimized nutritional management (see Appendix 3).

3) Enteral nutrition:

Our unit prioritizes oral feeding wherever feasible to enhance adaptation and minimize oral aversion. However, gastrostomy tubes are routinely placed for patients undergoing bowel lengthening procedures. These allow for overnight feeds, especially during growth spurts (e.g., around ages 3, 6, and puberty) when patients may otherwise fall off their growth curves.

### Data collection:

The electronic medical records were reviewed. The data collected included patient demographics, gestational age, corrected age, growth parameters such as weight in grams, the initial pathology, surgical intervention(s), operative findings, and remaining bowel length on initial and final surgery. The liver function tests were collected, use of PN, along with possible effects of PN, including cholestasis, IFALD, and central-line associated bloodstream infection (CLABSI).

### Statistical analysis

Descriptive statistics were used to summarize data, presented as number (percent) or median (interquartile range), as appropriate. We used the *t*-test (or Fischer's exact test) and Mann–Whitney test to examine factors associated with discontinuation of PN, with a *p*-value of < 0.05 considered statistically significant.

Weaning off PN, both overall and in association with the serial transverse enteroplasty procedure (STEP), was assessed using survival curves, with the median time to weaning for 50% of the cohort (T50) reported. Cox regression was applied to compare outcomes in patients who underwent STEP versus those who did not, with hazard ratios (HR) and 95% confidence intervals (CI) reported.

### Definitions

- ***Short bowel syndrome (SBS):**** Bowel length* less than 25% of the expected length for age at the time of initial surgery following intestinal resection, or any bowel resection with the requirement of parenteral nutrition (PN) for more than 60 days [4, 6, 7].
- ***Ultrashort SBS***: Bowel length less than 10% of the expected bowel length [6].
- ***PN-Cholestasis:*** A rise in serum-conjugated bilirubin of 2 mg/dL or more, which may be accompanied by increases in γ-glutamyl transpeptidase, alkaline phosphatase, and serum transaminase [22]
- ***Intestinal failure-associated liver disease (IFALD)*** describes liver injury, as manifested by cholestasis, steatosis, and fibrosis in patients with intestinal failure that is independent of, or in addition to, other potential etiologies [6].
- ***Central line-associated bloodstream infection (CLABSI)*** was defined as a positive blood culture with clinical signs of sepsis.
- ***Enteral autonomy*** enteral autonomy is the maintenance of normal growth and hydration status by means of enteral support without the use of parenteral support for a period greater than 3 consecutive months
- To correct for the gestation age, we used values such as: bowel length to gestational age in weeks (BL/A) and percent predicted bowel length (%PBL). The latter was calculated using bowel length estimates at different gestational ages from two key papers [12]. When provided a range, we chose the end (smallest or largest) closest to the patient’s age.

## Results

### Surgical cohort and group classification:

For the period of Jan 2018 to Feb 2024, 46 patients with SBS were followed at Sidra Medicine. Of these, 25 were premature with (< = 32 weeks gestation). After applying the exclusion criteria, 20 patients (10 males and 10 females) were included in the final analysis (see Appendix Table 1 for excluded).

The median gestational age was 28 weeks (range: 24–32 weeks) with a median birth weight of 860g (range: 540–1650g). The median age at onset of SBS (age at first surgery) was 32.42 corrected age in weeks gestation (range: 28–43.57 weeks). The primary cause of SBS was necrotizing enterocolitis (NEC) in 18 patients (90%), followed by volvulus in 1 patient (5%) and atresia in 1 patient (5%).

**Fig. 1:**
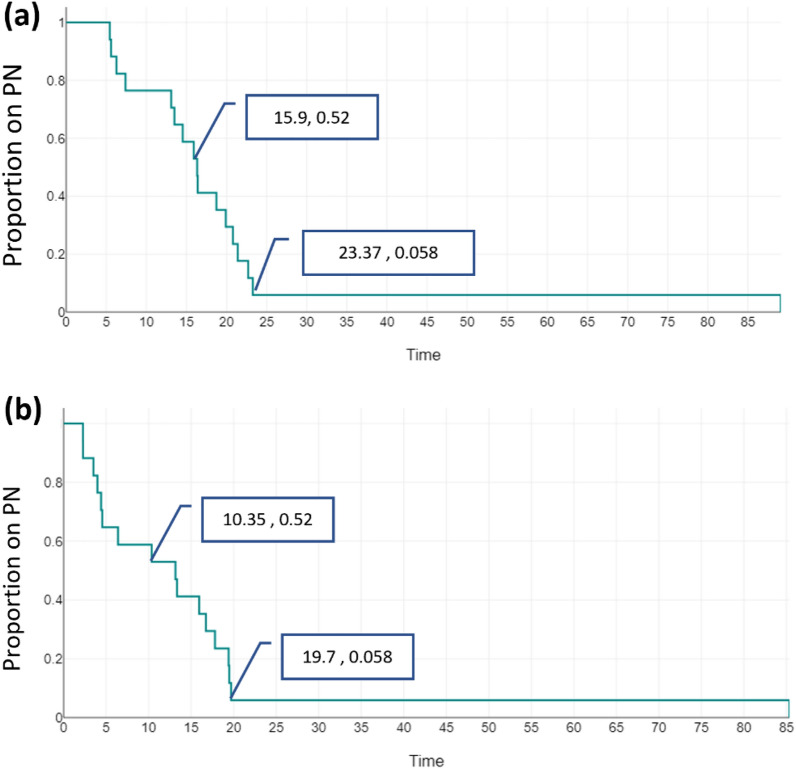
Survival curve showing overall weaning off PN based on actual age in months

**Fig. 2:**
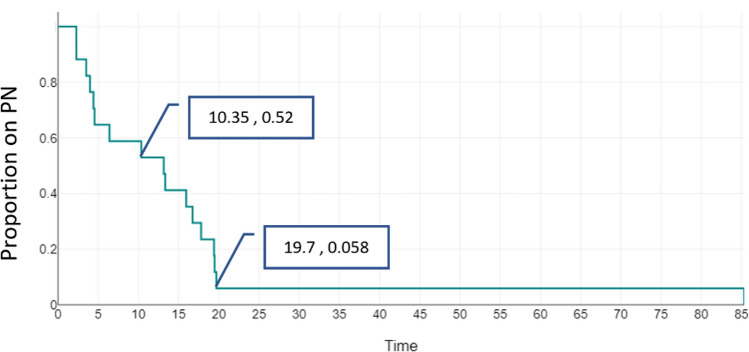
Survival curve showing overall weaning off PN based on corrected gestational age in months

A total of 16 patients (80%) were successfully weaned off parenteral nutrition (PN)—Group A, while 4 patients (20%) remained dependent on PN—Group B at the time of analysis (see Table 1). There were no statistically significant differences between the groups in terms of clinical factors such as gestational age, birth weight, or causes of SBS (Table 2). Bowel length on initial and final surgeries tended to be shorter for the group on PN. However, that was not statistically significant. Percent predicted bowel length (%PBL) was, similarly, not significantly different between group A and B at the two time points: onset and final surgery. There was no significant difference in the presence of ICV between groups A and B. The time to weaning for 50% of the cohort (T50) was 16.3 months of age (Figs. 1, 2).

**Table 1:** Comparison of bowel length, gestational age, and predicted bowel length between groups (expressed as median [IQR] or number [%])

Variable	GROUP A—off PN (*n* = 16)	GROUP B—on PN (*n* = 4)	*p*-value
Corrected age at first surgery (weeks)	31.43 (29.78–32.97)	34 (32.42–34.57)	0.331
Number of bowel related surgeries	2 (0.75–3)	2.5 (2–3.75)	0.554
Ileocecal valve (ICV) present	14/16 (87.5%)	3/4 (75%)	0.531
Initial bowel length (cm)	33 (22–51)	27.5 (15–39.25)	0.549
% of Predicted bowel length (Initial)	28.13 (24.83–42.22)	23.38 (11.49–39.88)	0.624
Final bowel length (cm)	60 (47.5–71)	35 (33–39)	0.197
% of Predicted bowel length (final)	25.08 (18.7–30.73)	14.63 (13.8–16.89)	0.142
# STEP (%)	6/16 (37.5%)	1/4 (25%)	0.693

**Table 2:** Baseline characteristics of patient population (expressed as median [IQR] or number [%])

Variable	*n* = 20	GROUP A (off PN) (*n* = 16)	GROUP B (on PN) (*n* = 4)
Gestational age at birth (weeks)	27.5 (range: 24–32)	27.5 (24.96–28.25)	28 (26.29–29.18)
Birth weight (grams)	860g (range: 540–1650)	850 (800.75–933.75)	945 (850–1047)
Gender: Male: Female	1:1	9:7	1:3
ICU stay (days)	187 (144–259.5)	194 (169–271.5)	132 (115.25–164.75)
Causes of SBS: NEC	18 (90%)	14/16 (87.5%)	4 (100%)
Central venous line (CVL) procedures (insertions, replacement)	5.5 (4.25–8.75)	6 (4.25–8.75)	5.5 (4.5–6.75)
Confirmed CLABSI episodes	2 (1–4)	3 (1–4.5)	2 (1.5–2.5)
PN cholestasis present	2/20 (10%)	0/16	2/4 (50%)
Deaths	2 (10%)	0	2
Age at last follow-up (corrected) months	49.7 (25–82.5)	57.67 (36.72–82.5)	13.5 (5.56–38.32)

Seven patients underwent STEP (35%), with 6/7 (85.7%) successfully weaned off PN at 17.57 months (IQR: 16.02–21.71). There was no statistically significant difference in initial bowel length between patients who had STEP and those that did not (*p* = 0.479). In STEP group 1/7 remain on PN, while 3/13 are on PN in the no-STEP group, two of which had died while on PN. This minimum number was reached by 23.27 months (19.7 months of corrected age). Demographics, age at onset of SBS, initial bowel length, %PBL, PN status and mortality were not significantly different between the two groups. Follow-up was shorter in group B compared to group A, with a median of 13.5 (5.56–38.32) and 57.67 (36.72–82.5), respectively.

### Complications and Mortality:

PN cholestasis occurred in 1/16 (6.25%) patients in group A, and in 3/4 (75%) patients in group B, a difference that was statistically significant (*p* < 0.0007). None of the patients required liver/bowel transplants. There were two moralities (10%): One patient had ultrashort bowel, developed IFALD and passed away with sepsis at 5.6 months; the other had multi-organ failure secondary to sepsis at 5.4 months. Both mortalities were receiving PN at the time of death (included under group B).

### Follow-up

There were no patients lost to follow-up in this series. Last follow-up was at median age 49.7 (IQR 25–82.5), and this was similar for groups A and B.

## Discussion

Enteral autonomy is the ultimate goal in SBS patients. Several factors increase the likelihood of weaning from parenteral nutrition (PN), including a diagnosis of necrotizing enterocolitis (NEC), a larger proportion of intact residual colon, the presence of an intact ileocecal valve, and especially residual bowel remains a very important predictor of intestinal autonomy [14]. There is more evidence showing that care provided at a specialized non-transplant intestinal rehabilitation center has a tremendous impact on outcomes [13]. These factors contribute to improved outcomes in neonates compared to older children with SBS.

According to the American Society for Parenteral and Enteral Nutrition (ASPEN) pediatric intestinal failure (PIF) working group, short-bowel syndrome (SBS) is considered a subcategory of intestinal failure (IF) caused by an overall loss of small-bowel length, whether due to resection or congenital absence [6]. This definition and various others have emphasized two key aspects: a shortened length of the small intestine and a need for prolonged parenteral nutrition (PN). Various organizations and experts have proposed different definitions for prolonged PN use. For instance, the Canadian Association of Pediatric Surgeons defined SBS as either the need for PN lasting more than 42 days after bowel resection and / or a residual small bowel length of less than 25% of the expected length for gestational age [4]. This is the definition we have adopted at Sidra Medicine. In addition, ultrashort-bowel syndrome is defined as the development of IF following significant small-bowel resection resulting in a residual small-bowel length that is less than 10% of expected [6].

Length-based definitions of short-bowel syndrome (SBS) are often inadequate due to the significant variation in bowel length across different gestational ages and the rapid growth of the bowel during this time. There is rapid bowel growth during early gestation and the first year of life, making precise bowel measurements difficult using available information [10–12]. One of the early studies by Robert et al. on stillborn infants showed that between 19- and 35-weeks’ gestation, bowel lengths ranged from 142 to 304 cm [11]. A landmark study by Marie-Chantal et al., measured bowel lengths intraoperatively in 108 living neonates, provided the ranges of normal bowel length for the examined gestational ages [12]. The most recent study by Bardwell et. Al established ranges for expected small and large bowel length in fetal and neonatal bowel, with average and 90% CI for lower limit, and average and 90% CI for upper limit for gestational age [10]. All the literature available shows wide variation of “predicted length”, and does not allow for precise length prediction according to gestational age. These findings highlight the need for more individualized prognostic tools that account for gestational age.

. Creating more precise reference intervals (RIs) for estimated bowel length and subsequent calculation of percent of predicted bowel length will likely improve diagnosis, surgical planning, and nutritional management, particularly for preterm infants who are at higher risk for intestinal complications. These nomograms will enable accurate prognostication, guide surgical decisions, and facilitate tailored nutritional strategies to promote bowel adaptation and reduce dependence on parenteral nutrition. Studies show that lower birth weight correlates with a higher incidence of intestinal failure: infants weighing between 401 and 1000g had an incidence of 1.1%, nearly double that of those weighing 1001–1500g [3]. Similarly, preterm infants (< 37 weeks) experience higher incidences of SBS than full-term infants, with a significantly higher mortality rate of 37.5% [17]. Our study showed a trend of slower weaning from PN among older infants at SBS onset, with smaller bowel growth between onset and final surgery compared to younger infants, although this difference was not statistically significant. In our cohort, we used several metrics—absolute bowel length and percentage of predicted bowel length. But none showed a statistically significant association with the need for prolonged PN.

Interestingly, infants with NEC, a common cause of SBS, often have better outcomes than those with SBS from other causes [23]. NEC accounted for 80% of the population in our study. Improved outcomes in NEC may be due to the timing of NEC's onset, which coincides with periods of rapid bowel growth in preterm infants, possibly aiding adaptation. This observation warrants further research to optimize care strategies for preterm infants with SBS.

### Intestinal autonomy

According to 2007 data from the pediatric intestinal failure consortium, which tracked 272 infants, the reported rates were 47% for enteral autonomy, 27% for mortality, and 26% for intestinal transplantation [15]. However, outcomes for children with intestinal failure have improved significantly since then. Advances in hepatoprotective PN, widespread adoption of mixed lipid formulations, and the establishment of specialized multidisciplinary intestinal rehabilitation units have transformed the prognosis. As a result, the need for intestinal transplantation (ITx) has decreased, with transplants now reserved only for highly selective cases.

A 2018 study by Norsa et al. identified two critical periods for weaning off TPN: between 2 and 4 years of age and around puberty (16–18 years). The findings showed that even children with extremely short bowel lengths (< 10 cm) could achieve intestinal sufficiency through structured management, emphasizing the importance of making systematic weaning attempts before considering ITx [16]. Similarly, a 2011 multicenter cohort study by Pironi et al. reported a 24% weaning rate, and another study focused on ultra-short bowel syndrome (USBS) found that 48% of affected children were successfully weaned off TPN. These results underscore the potential of aggressive intestinal rehabilitation in specialized centers to improve outcomes.

In our cohort, while we still lack long-term outcomes, promising progress has been observed. Out of 20 patients on PN during the 2–4-year window, six have been successfully weaned off PN. Unfortunately, two patients passed away while on PN, but 3/4 currently on PN are in the process of weaning, now only requiring PN few nights per week. Weaning is closely managed in our multidisciplinary clinic, with careful monitoring for overfeeding signs and regular weight tracking to ensure safe and sustainable progress toward enteral autonomy. Our multi-disciplinary care helped achieve a great rate of PN weaning of this premature cohort especially when looking at the corrected age, majority achieving enteral autonomy by 19.7 months.

### Mortality

Mortality rates in neonates with intestinal failure (IF) and SBS vary significantly, with outcomes influenced by early complications and long-term disease management. Cole et al. studied VLBW neonates with varying age groups and showed Infants with surgical NEC without SBS exhibit the highest in-hospital mortality (55%) and lowest long-term survival (43% at 180 days) compared to other groups, reflecting the critical severity of surgical NEC. In contrast, SBS infants show better initial survival (79% at 180 days) but face significant challenges, such as delayed achievement of full enteral feeds (66% within 120 days) and higher post-discharge mortality (10%), necessitating early interventions and specialized care strategies [3]. Mortality in SBS follows a bimodal pattern—early deaths occur from surgical and disease-related complications, while long-term mortality arises from intestinal failure-associated liver disease (IFALD) and sepsis, particularly catheter-associated bloodstream infections (CABSI) [13]. Fullerton et al. collected data from a variety of single centers and the Pediatric Intestinal Failure Consortium (PIFCON), which studied 14 North American intestinal rehabilitation centers, showing that with multidisciplinary care, long-term survival in pediatric IF exceeds 90% [13]. Additionally, retrospective reviews from large intestinal transplant centers highlight the impact of parenteral nutrition (PN) dependence on mortality: five-year survival is 95% for SBS patients weaned from PN, compared to 52% in those remaining on PN [17, 18]. These findings emphasize the importance of multidisciplinary intestinal rehabilitation programs and hepatoprotective strategies in PN management for improving outcomes in these vulnerable populations. We lost 2 patients in our series of 20 neonates (10% mortality). Both were secondary to sepsis at the ages of 5.4 and 5.6 months of corrected age.

### Serial transverse enteroplasty (STEP) and intestinal autonomy

Since its introduction by Kim et al., the serial transverse enteroplasty (STEP) procedure has become a pivotal approach in the management of short bowel syndrome (SBS). Primarily used for bowel lengthening, STEP also addresses bacterial overgrowth, improves nutrient absorption, and promotes intestinal adaptation. Studies involving both children and adults report intestinal autonomy rates ranging from 47 to 69%, indicating that many patients can successfully wean off PN [19–22]. Even among those who remain dependent on PN, STEP often reduces the volume and frequency of PN required, facilitating cycling strategies that improve quality of life and reduce long-term risks associated with continuous infusion.

A recent multicenter study involving 36 children who underwent the STEP procedure demonstrated a PN weaning rate of 42%. However, the study also identified two major prognostic factors limiting PN independence: bowel length under 40 cm at initial surgery and absence of the ileocecal valve, both of which significantly impact the potential for achieving full intestinal autonomy [20]. In our study, 7 of 20 patients underwent STEP.

This study was limited by its retrospective nature and small sample size. Many of the conclusions drawn through this paper would have to be verified with a bigger sample size and potentially multi-center collaboration. However, it represents the largest reported cohort in the literature for premature babies with SBS. Additionally, our local treatment protocols have evolved over time, potentially influencing the outcomes observed.

## Conclusion and future directions

This is the largest published series with premature babies with SBS from a single center. Our experience shows that this cohort has comparable survival and native liver survival compared to the published literature. The rate of PN weaning is comparable to other cohorts despite prematurity, which may show a higher capacity for adaptation and growth in these premature infants. The PN weaning rate is suggested to be better at an earlier onset of SBS. Future studies to investigate that aspect as well as the role gestational age in general in PN weaning. Most importantly, a multi-disciplinary approach is essential for achieving such results.

Further research is needed to establish gestational-age-based nomograms for bowel length, as traditional approximations are insufficient for managing neonatal short bowel syndrome (SBS).

Large multicenter collaborations are required to better characterize reference intervals for normal bowel, and its importance in the management algorithm of SBS patients. The use of advanced technologies like AI can further refine these metrics, helping clinicians optimize long-term outcomes for infants with SBS. (Table 3)

**Table 3:** Comparison of STEP and no-STEP groups in demographics and outcomes (expressed as median [IQR] or number [%])

	STEP (*n* = 7)	No STEP (*n* = 13)	*p*
Gestational age at birth (wks)^a^	27 (24–32)	28 (24.14–32)	0.311
Birth weight (g)^a^	880 (671–1650)	850 (540–1130)	0.438
Gender			
Male	4	6	
Female	4	6	1
Age at onset of SBS (coorected age in weeks)	33 (29.79–34.57)	31.85 (29.71–32.86)	0.588
Initial bowel length (cm)	30 (23–53.25)	36 (18.75–34)	0.27
% of predicted bowel length (Initial)	26.32 (18.83–31.48)	35.15 (19.41–48.92)	0.27
Bowel length on final surgery (cm)	72 (59.5–79)	46 (35–60)	0.056
% of predicted bowel length on final surgery	30.1 (22.57–33.03)	16.92 (14.63–25.99)	0.183
Currently on PN	1	3	0.494
Mortality	0	2	0.224

^a^Median and range

## Data Availability

Data is provided within the manuscript. Any additional data may be provided when requested.

## Abbreviations

SBS: Short bowel syndrome
PN: Parenteral nutrition
BEAR: Bowel expansion and rehabilitation
STEP: Serial transverse enteroplasty
VLBW: Very low birth weight

## Declarations

### Appendix

See Table 4,5,6

**Table 4:** Characteristics of excluded patients

#	Gestational age at birth (wks.)	Gender	Cause of SBS
1	25	M	Complicated meconium ileus
2	26	M	Intestinal atresia
3	30.85	M	NEC
4	24	F	NEC
5	23.14	F	NEC

**Table 5:** Case by case operative findings and PN status.

Patinet no	Length of remaining small bowel at initial surgery	Lengthened?	Length of remaining small bowel at final surgery	Weaned from PN?
1	33	No	53	Yes
2	35	Yes	69	Yes
3	12	No	33	Yes
4	9	Yes	30	No
5	60	No	?	Yes
6	29	Yes	49	Yes
7	32	No	35	Yes
8	22	Yes	72	Yes
9	15.5	Yes	70	Yes
10	51	No	60	Yes
11	56	No	80	Yes
12	15	No	25	No
13	35	No	46	Yes
14	5	No	Expired	No
15	16	No	35	No
16	54	No	105	No
17	39	No	Expired	No
18	20	No	70	Yes
19	40	Yes	180	Yes
20	33	Yes	75	Yes

**Table 6:** PN management strategies to limit risk of IFALD

Aspect	Details
Lipid policies	Lipid used: SMOFlipid for all patients Omegaven: used when bilirubin and liver enzymes are persistently high despite reducing/withholding lipids
Lipid dosing	• Initial lipid infusion • Preterm and term infants: Start at 1–1.5 g/kg/day • Maximum dose: o Standard infants: do not exceed 3 g/kg/day o High-risk infants (e.g., those at risk for IFALD): Limit to ≤ 2.5 g/kg/day • Lipid management for IFALD (Intestinal Failure-Associated Liver Disease) • Mild to moderate IFALD: reduce lipids to the minimum required to meet essential fatty acid (EFA) needs (1–1.5 g/kg/day) • Severe or refractory IFALD: if bilirubin levels remain elevated despite lipid restriction, consider complete lipid discontinuation until liver function stabilizes
Protein policies	Initiate at 2 g/kg/day on Day 1 for preterm infants and 1.5–2 g/kg/day for term infants Target: 2.5–3.5 g/kg/day (max 4 g/kg/day for preterm)
Carbohydrate policies	1. Initial dextrose infusion (day 1) • Preterm infants: start with a GIR of 4–8 mg/kg/min (5.8–11.5 g/kg/day) • Term infants: start with a GIR of 2.5–5 mg/kg/min (3.6–7.2 g/kg/day) 2. Gradual increase from day 2 onward • Preterm infants: increase GIR to 8–10 mg/kg/min (11.5–14.4 g/kg/day) as tolerated • Term infants: increase GIR up to 5–10 mg/kg/min (7.2–14.4 g/kg/day)
IFALD management	Strategies include: - Early enteral feeding (trophic feeds when possible) - SMOFlipid as the first-line lipid - Consider omegaven if cholestasis persists despite interventions
Oral feeding vs. NGT/gastrostomy	Oral feeding is prioritized when feasible Gastrostomy tubes are routinely placed in patients undergoing bowel lengthening or with persistent oral aversion
Monitoring	Regularly monitor bilirubin, liver enzymes, electrolytes, and growth parameters Use cyclical PN as tolerated to support liver function
